# Serum Trimethylamine N-Oxide Level Is Associated with Peripheral Arterial Stiffness in Advanced Non-Dialysis Chronic Kidney Disease Patients

**DOI:** 10.3390/toxins14080526

**Published:** 2022-07-31

**Authors:** Bang-Gee Hsu, Chih-Hsien Wang, Yu-Li Lin, Yu-Hsien Lai, Jen-Pi Tsai

**Affiliations:** 1 Division of Nephrology, Department of Internal Medicine, Hualien Tzu Chi Hospital, Buddhist Tzu Chi Medical Foundation, Hualien 97010, Taiwan; gee.lily@msa.hinet.net (B.-G.H.); wangch33@gmail.com (C.-H.W.); nomo8931126@gmail.com (Y.-L.L.); hsienhsien@gmail.com (Y.-H.L.); 2 School of Medicine, Tzu Chi University, Hualien 97004, Taiwan; 3 Division of Nephrology, Department of Internal Medicine, Dalin Tzu Chi Hospital, Buddhist Tzu Chi Medical Foundation, Chiayi 62247, Taiwan

**Keywords:** brachial-ankle pulse wave velocity, chronic kidney disease, peripheral artery stiffness, trimethylamine N-oxide

## Abstract

Trimethylamine N-oxide (TMAO) is a gut-derived uremic toxin involved in cardiovascular diseases (CVD). Peripheral arterial stiffness (PAS), measured by the brachial-ankle pulse wave velocity (baPWV) is a valuable indicator of the existence of CVD alongside other diseases. The study recruited 157 patients with chronic kidney disease (CKD) stages 3 to 5, and aimed to determine the correlation between serum TMAO and PAS, defined as a baPWV of >18.0 m/s. Patients with CKD who were diagnosed with PAS (68 patients, 43.3%) were older, had a higher percentage of hypertension or diabetes mellitus, higher systolic blood pressure, and higher fasting glucose, C-reactive protein, and TMAO levels. Furthermore, besides old age and SBP, patients with CKD who had higher serum TMAO were more likely to have PAS, with an odds ratio of 1.016 (95% confidence interval = 1.002–1.029, *p* = 0.021) by multivariate logistic regression analysis. Correlation analysis demonstrated that serum TMAO was positively correlated with C-reactive protein level and either left or right baPWV. Thus, we supposed that serum TMAO levels were associated with PAS in patients with advanced non-dialysis CKD.

## 1. Introduction

Patients with chronic kidney disease (CKD) were found to accumulate all kinds of waste products, including noxious metabolic by-products absorbed via the intestines as the kidneys lost their excretion abilities [1]. Existing evidence demonstrated that gut-derived metabolic harmful products could induce adverse effects such as cardiovascular diseases (CVD) and even CKD progression. Indoxyl sulfate and p-cresol sulfate, two well-known protein-bound uremic toxins, were produced from the intestinal bacterial flora, and could be associated with CKD progression and future CVD development in non-dialytic or dialytic CKD [2,3,4]. Similarly, trimethylamine N-oxide (TMAO), also known as another kind of gut microbiota-derived metabolite, originated from trimethylamine [1]. Over the past decade, TMAO had been implicated to have an important role in the occurrence of various diseases, including diabetes mellitus, hypertension, heart failure, inflammation, and most importantly, CKD development [5,6,7,8]. Previous studies reported that TMAO is physiologically filtrated and secreted unchanged by the kidneys and would accumulate as renal function worsened. The high TMAO concentration could, in turn, accelerate kidney dysfunction progression by modulating the progress of tubule-interstitial fibrosis and collagen deposition [9,10,11,12]. An animal study demonstrated that the TMAO of rats that underwent 5/6 nephrectomy could induce vascular oxidative stress and inflammation, resulting in endothelial dysfunction and further CVD [13]. Furthermore, evidence from meta-analysis and systemic review had shown that TMAO correlated with a higher risk of hypertension [14] and all-cause and adverse CV-associated events in the general population [15], geriatric population [16], and patients with CKD [17].

Previous studies have shown that vascular stiffening, as measured by pulse wave velocity (PWV), was correlated with CVD, which was long recognized as the main cause of long-term adverse outcomes in CKD through the possible mechanisms of oxidative stress, low-grade inflammation, or disordered mineral metabolism [18,19,20]. In this study, we measured peripheral arterial stiffness (PAS) by applying a noninvasive modality as brachial-ankle PWV (baPWV), which could be an indicator of CKD progression or mortality, to assess the association with the serum TMAO in patients with non-dialysis advanced CKD [21]. As we had reported, the gut-derived uremic toxins, indoxyl sulfate, and p-cresol sulfate, were correlated with endothelial dysfunction and arterial stiffness [22,23,24], we hypothesized that TMAO could have a potential role in the occurrence of peripheral arterial stiffness. 

## 2. Results

A total of 68 (43.3%) and 89 (56.7%) patients were categorized in the PAS and control groups (Table 1). Compared to the control group, patients in the PAS group were older, had higher fasting glucose, systolic blood pressure (SBP), percentage of DM and HTN, and serum C-reactive protein (CRP) and TMAO levels. Patients with CKD in the PAS group had a lower estimated glomerular filtration rate (eGFR) compared to those in the control group (*p* = 0.001); however, when classifying patients into CKD stages, no significant difference was observed between those in the PAS and control groups (*p* = 0.019). Viewed more closely, a trend could be observed showing a higher percentage of CKD stages 4 and 5 in patients belonging to the PAS group compared to the control group (chi-squared test for trend, *p* = 0.044, data not shown). 

We applied multivariate logistic regression analysis on age, HTN, DM, SBP, fasting glucose, eGFR, CRP, and TMAO to analyze possible factors indicative of PAS occurrence. It showed that patients with CKD who were older had higher SBP and serum TMAO, with a 1.081-fold (95% confidence interval [CI] = 1.043–1.121, *p* < 0.001), 1.042-fold (95% CI = 1.004–1.081, *p* = 0.031), and 1.016-fold (95% CI = 1.002–1.029, *p* = 0.021) increased risk of PAS occurrence (Table 2).

**Figure 1:**
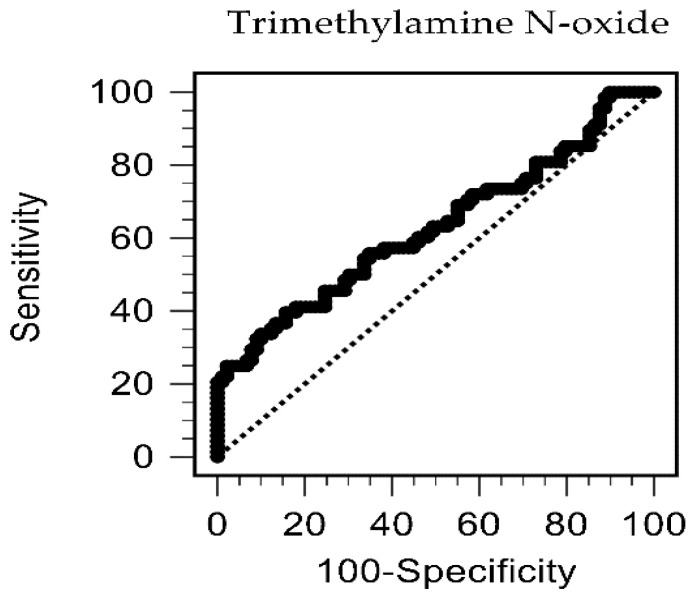
Levels of TMAO relative to the occurrence of peripheral arterial stiffness in chronic kidney disease patients by receiver operating characteristic curve analysis.

To determine the serum TMAO level relative to PAS occurrence, a receiver operating characteristic curve analysis was applied, which showed an optimal serum cut-off level of 46.78 μg/L with 39.71% and 84.27% in sensitivity and specificity, individually. The area under the curve was 0.629 (95% CI = 0.548–0.705, *p* = 0.0052, Figure 1).

Table 3 shows the correlation between clinical variables and baPWV [left and right] and serum TMAO by Spearman correlation analysis. First, left baPWV was strongly correlated with right baPWV (*r* = 0.913, *p* < 0.001). Second, both left and right baPWV were significantly positively correlated with aging, SBP, and serum TMAO. Third, in addition to the positive association with baPWV, serum TMAO level was found to be positively correlated with aging (*r* = 0.219, *p* = 0.006) and CRP (*r* = 0.261, *p* = 0.001), but negatively associated with eGFR (*r* = −0.442, *p* < 0.001).

To examine the potential risk of TAMO related with PAS in CKD patients, a subgroup analysis was performed by dividing patients into groups with or without DM, HTN, and hyperlipidemia (Table 4). The analysis revealed that a higher TMAO value significantly correlated with the occurrence of PAS specifically in CKD patients who were without DM, and those with or without HTN or hyperlipidemia. TMAO in CKD stage 4 and 5 patients had an increasingly independently 1.025-fold (*p* = 0.046), and 1.036-fold (*p* = 0.021) higher risk of developing PAS, respectively. It indicated that there was increased possibility of TMAO in relation to the occurrence of PAS in advanced CKD. 

## 3. Discussion

In this study, TMAO associated with aging and high SBP could be a useful marker for the occurrence of PAS in patients with advanced CKD. Furthermore, serum TMAO increased as patients got older, exhibited a greater inflammatory status, or exhibited a decline of renal function.

CVD has been known to be the leading cause of long-term morbidity or mortality in patients with dialytic or non-dialytic CKD [18]. In this study, aging and high SBP were found to be associated with PAS, as indicated by baPWV. As is already known, many traditional and uremia-specific risk factors could lead to irreversible vascular damage with results in high vascular smooth muscle tone and BP, as manifested in arterial stiffness [19,20]. Previous studies had shown that old age and high BP contributed to AS [25,26], which could, in turn, promote further CVD development, renal dysfunction, and even mortality [19,20]. Furthermore, a recent study showed that with old age and SBP, TMAO was also associated with increased PWV in mice as well as in healthy young- to old-aged adults [27].

In addition to the traditional risk factors, such as old age or high BP (SBP or DBP), gut-derived uremic toxins, such as indoxyl sulfate, p-cresol sulfate, and TMAO, were demonstrated to play important roles in CVD pathogenesis [2,3,4]. Through oxidative stress modulation and inflammatory and cytokine responses, which then induced endothelium and smooth muscle cell dysfunctions, these gut-derived uremic toxins were found to have detrimental effects on vascular health [28,29]. Our previous studies also showed that indoxyl sulfate and p-cresol sulfate were related to the occurrence of arterial stiffness in patients with CKD, which highlighted the important pathogenetic role of gut-derived uremic toxins on AS [22,23,24]. Furthermore, TMA, produced from dietary choline, phosphatidylcholine, L-carnitine, and betaine by intestinal bacteria, and then converted to TMAO in the liver, was accumulated in patients with CKD [1]. Besides, serum TMAO was significantly elevated in association with DM, age, and BMI [6], and was positively dose-dependently associated with HTN, as increased levels of per-5 mμmol/L or 10-mμmol/L would increase the risk of HTN by 9% and 20%, respectively [30]. In animal studies, increased dietary TMAO would induce tubule-interstitial fibrosis, collagen deposition, and Smad3 phosphorylation, and then renal function indicated increased serum cystatin C [9]. Another animal study also showed that TMAO-activated inflammasomes release interleukin-1 beta and interleukin-18 to accelerate renal inflammation and fibrosis [10]. Conversely, after targeting TAMO inhibition, it attenuated serum cystatin C levels as well as renal tubule-interstitial fibrosis severity and collagen deposition [11]. In a meta-analysis, patients with advanced CKD had a 67.9 mμmol/L increase in TMAO concentration, whereas patients with CKD had the highest TMAO level of 12.9 mmol/L decreases in eGFR [12]. In regard to renal function, elevated TMAO could only return to normal range after receiving renal transplantation without receiving dialysis [31]. Farhangi et al. found a positively dose-dependent nonlinear relationship between serum TMAO and CRP (the highest category had a 0.27 mg/L increase in CRP compared to the lowest) [8]. Because TMAO was a metabolite derived from gut microbial metabolism and dietary intake, Mishima et al. found that TMAO could originate from both microbiota and dietary components [32]; Costabile et al. found that marine derived diets induce higher TMAO in a healthy population [33]; in a randomized crossover designed study comparing plant-based alternative meat to animal meat, a significant lower level of TMAO was observed in a healthy population on plant-based alternative meat [34]; another study showed a 2-month vegan diet could significantly reduce plasma TMAO in obese or hyperglycemic adults [35]; but a meta-analysis comparing microbiota-driven therapy, including prebiotics, probiotics or synbiotics, with placebo did not reveal a reduction in circulating TMAO [36]. Although we did not record the dietary contents of CKD patients in this study, we still found that, as already shown, there was an inverse relationship between serum TMAO and kidney function and a positive correlation between TMAO and CRP.

Aside from the harmful effects on renal function, many studies reported the TMAO effects on CV and long-term adverse outcomes. Evidence had shown an association between TMAO and severity and mortality in patients with heart failure [5]. Tarng et al. conducted a cohort study on patients with stage 3 to 5 CKD, with a prognostic value of TMAO in predicting 5-year mortality risk [9]. Several meta-analyses showed that TMAO had a dose-dependent effect relationship with increased risk of HTN (the highest category had a 2.36 mmHg increase in SBP compared to the lowest) [14], an increased risk of all-cause mortality and CV events in the general and elderly population [15]. In patients with CKD with the highest TMAO group, a 1.29-fold and 1.45-fold increased risk was observed of all-cause and CV mortality; besides, a 3% higher all-cause mortality per 1 unit increases the TMAO level [17]. Studies have proven that TMAO plays a role in the atherosclerosis process through mediating immune response by recruiting macrophages to aortic lesions with increased CD36, tumor necrotic factor α, and interleukin-6 expressions through the p38 MAPK and JNK 1/2 pathways [37], or activation of NLRP3 inflammasome to cause endothelial dysfunction and inflammation through redox regulation and lysosomal dysfunction [38]. Furthermore, endothelial and smooth muscle cells exposed to TMAO enhanced the recruitment of leukocytes and induced various inflammatory cytokines by activating nuclear factor-kappa β [39]. In 5/6 nephrectomy rats, TMAO with superoxide and proinflammatory cytokines were found to be significantly elevated but with reduced production of endothelial nitric oxide, which together contributed to endothelial dysfunction [13]. Most importantly, TMAO was recently found to modulate the vascular calcification process [40]. Zhang et al. found that TMAO could upregulate osteoblast-specific protein expression, such as Runt-related transcription factor 2 and bone morphogenetic protein-2, to induce calcification by activating nuclear factor-kappa β and NLRP3 inflammasomes [40]. Brunt et al. conducted a study and showed plasma TMAO with aging positively correlated with aortic PWV and SBP in humans and mice; in vitro study additionally revealed the adverse effects of TMAO on intrinsic mechanical stiffness by modulating the formation of advanced glycation end-products and reactive oxygen species [27]. Additionally, this study showed interesting findings that TMAO levels for those CKD patients who did not have DM, regardless of HTN or hyperlipidemia, showed a significantly higher risk for the occurrence of PAS. However, the TMAO of diabetic CKD patients did not correlate with the occurrence of PAS by multivariate logistic regression analysis. This result showed the possible role of DM for influencing the role of TMAO on PAS. Taken together, we reported a positive correlation between serum TMAO level and baPWV in patients with advanced CKD, with a possible mechanism from inflammatory activations and DM influencing the pathogenesis of TMAO on PAS.

This study had several limitations. First, it was a cross-sectional and single-center design with a limited sample size of patients with CKD. Furthermore, patients with stage 1–2 CKD and on dialysis were excluded. Therefore, the results could not be extrapolated to other populations, except for advanced CKD. Second, gut microbiota was not assessed in this study, and the mechanism by which TMAO affects PAS remains unclear. Third, the association between TMAO and PAS (baPWV) was not stronger than age and SBP. Nevertheless, we thought that there could be a role of TMAO in the development of PAS, probably through the activation of inflammatory responses. Fourth, we did not record the dietary contents of these CKD patients. Indeed, plasma TMAO still presents in animals without microbiota [32], and the level directly correlates with the intake of fish, vegetables, and whole-grain products, but not meat, processed meat, and dairy products in humans with high cardiometabolic risk [33]. Therefore, further longitudinal studies with large samples and cells as well as animal studies are needed to clarify these findings.

## 4. Conclusions

In addition to older age and elevated SBP, increased serum TMAO was independently indicative of PAS in patients with stage 3–5 CKD. Besides the positive correlation with baPWV, serum TMAO was also positively associated with serum CRP levels. These findings suggested that TMAO may be an important upstream modulator of the PAS development process among patients with advanced CKD who are diagnosed with stage 3–5 CKD, but the detailed mechanism remains to be investigated.

## 5. Materials and Methods

### 5.1. Patients

From January to December 2016, patients with CKD who were diagnosed with stage 3 to 5 CKD and were old than 18 years with regular follow-up were recruited. CKD was defined using the eGFR by the Chronic Kidney Disease Epidemiology Collaboration equation [18]. Patients were separated into stages 3, 4, and 5 based on their calculated eGFRs of 30–59 mL/min/1.73 m^2^, 15–29 mL/min/1.73 m^2^, and <15 mL/min/1.73 m^2^ [18]. Patients with acute kidney injury, malignancies, acute infectious diseases, congestive heart failure at the time of blood sampling, or who refused to participate in this study were excluded. This study was approved by the Research Ethics Committee of Hualien Tzu Chi Hospital, Buddhist Tzu Chi Medical Foundation (IRB108-96-B).

The demographic and baseline characteristics regarding the smoking status, DM, HTN, and use of medications were recorded by medical record review.

### 5.2. Anthropometric and Biochemical Examination

Body height and weight were examined to the nearest 0.5 cm and 0.5 kg and BMI was calculated as weight (kg) divided by height squared (m^2^). The serum fasting glucose, blood urea nitrogen, creatinine, albumin, total cholesterol, triglyceride, low-density lipoprotein cholesterol, blood urea nitrogen, creatinine, and CRP levels were determined using an autoanalyzer (Siemens Advia 1800; Siemens Healthcare GmbH, Henkestr, Erlangen, Germany) after overnight fasting for at least 8 h.

### 5.3. Determination of Serum TMAO Levels by High-Performance Liquid Chromatography–Mass Spectrometry

We used a Waters e2695 high-performance liquid chromatography system containing a mass spectrometer (ACQUITY QDa, Waters Corporation, Milford, MA, USA) to determine serum TMAO levels according to a previous study [41]. Mass spectrometry was performed in full scan ranges of 50–450 m/z for positive-ion modes and 100–350 m/z for negative-ion modes, respectively, to monitor the participants’ compounds (TMAO: 76.0 m/z; d_9_-TMAO: 85.1 m/z). The retention time for TMAO and d_9_-TMAO was 2.54 min. The procurement and analysis of all examinations were performed using the Empower^®^ 3.0 software (New York, NY, USA).

### 5.4. Examination of Left and Right Brachial-Ankle PWV

Using this apparatus for volume plethysmography (VaSera VS-1000, Fukuda Denshi Co. Ltd., Tokyo, Japan), patients lay in a supine position with four pneumatic cuffs wrapped around both upper arms and ankles, connected to both plethysmographic and oscillometric sensors to assess baPWV after blood sampling, and rested for 10 min [25,26]. We used baPWV of >18 m/s (either left or right) as a cut-off value to diagnose PAS [25,26].

### 5.5. Statistical Analysis

The Kolmogorov–Smirnov test was used to examine the normality of continuous variables. The results indicated that these continuous data were expressed as the mean ± standard deviation or median with an interquartile range. To compare the difference of continuous variables between the control and PAS groups, the Student’s independent *t*-test or Mann–Whitney *μ*-test were used for the Kolmogorov–Smirnov test, as appropriate. Numbers with percentages and compared between categorical variables were calculated using the chi-squared test. Factors showing significant differences in Table 1 were adopted for multivariate logistic regression analysis, including DM, HTN, age, SBP, fasting glucose, eGFR, CRP, and TMAO, to determine possible factors associated with the occurrence of PAS (which is defined as baPWV as >18.0 m/s). A receiver operating characteristic curve analysis was used to determine the optimal serum TMAO levels indicative of PAS occurrence. The Spearman rank correlation coefficient was used to analyze the correlation between clinical variables and baPWV (left and right) and TMAO. Subgroup analysis by univariate logistic regression analysis was applied to analyze the relation between the occurrence of PAS and risk factors of CKD, including CKD stage, and underlying diseases such as DM, HTN, and hyperlipidemia. The SPSS software for Windows (version 19.0; SPSS, Chicago, IL, USA) was used for all statistical analyses. A *p* < 0.05 was indicative of statistical significance.

## Figures and Tables

**Table 1:** Baseline characteristics of the 157 chronic kidney disease patients in the control and peripheral arterial stiffness group.

Characteristics	All Patients (*n* = 157)	Control group (*n* = 89)	PAS Group (*n* = 68)	*p* Value
Age (years)	67.90 ± 13.00	63.35 ± 12.04	73.85 ± 11.81	<0.001 *
Body mass index (kg/m^2^)	26.12 ± 3.95	26.27 ± 4.39	25.92 ± 3.30	0.587
Female, *n* (%)	76 (48.4)	41 (46.1)	35 (51.5)	0.502
TMAO (μg/L)	27.43 (16.68–49.54)	25.28 (15.56–42.47)	35.83 (17.95–77.79)	0.006 *
Left baPWV (m/s)	17.30 ± 3.46	14.93 ± 1.54	20.40 ± 2.74	<0.001 *
Right baPWV (m/s)	17.26 ± 3.39	14.97 ± 1.80	20.26 ± 2.54	<0.001 *
Diabetes mellitus, *n* (%)	52 (33.1)	23 (25.8)	29 (42.6)	0.027 *
Hypertension, *n* (%)	97 (61.8)	49 (55.1)	48 (70.6)	0.047 *
Hyperlipidemia, *n* (%)	93 (59.2)	55 (61.8)	38 (55.9)	0.555
Smoking, *n* (%)	16 (10.2)	10 (11.2)	6 (8.8)	0.244
SBP (mmHg)	144.31 ± 17.84	140.53 ± 19.49	149.25 ± 14.09	0.002 *
DBP (mmHg)	82.14 ± 10.21	82.06 ± 10.31	82.25 ± 10.15	0.907
Total cholesterol (mg/dL)	163.01 ± 43.75	165.22 ± 47.37	160.12 ± 38.64	0.470
Triglyceride (mg/dL)	121.0 (87.5–165.0)	116.0 (88.5–166.5)	128.50 (86.25–163.0)	0.430
LDL–C (mg/dL)	92.63 ± 36.28	95.93 ± 39.41	88.31 ± 31.50	0.193
Fasting glucose (mg/dL)	104.0 (98.0–134.0)	100.0 (97.0–118.5)	110.0 (100.0–141.75)	0.010 *
Blood urea nitrogen (mg/dL)	33.00 (24.00–45.00)	31.00 (24.50–44.00)	35.50 (24.00–49.50)	0.562
Creatinine (mg/dL)	1.90 (1.40–2.70)	1.80 (1.40–2.60)	2.05 (1.60–3.00)	0.163
eGFR (mL/min)	31.37 ± 15.33	34.06 ± 15.42	27.84 ± 14.58	0.011 *
CKD stage 3, *n* (%)	79 (50.3)	51 (57.3)	28 (41.2)	0.119
CKD stage 4, *n* (%)	45 (28.7)	23 (25.8)	22 (32.4)	
CKD stage 5, *n* (%)	33 (21.0)	15 (16.9)	18 (26.4)	
Albumin (g/dL)	4.07 ± 0.32	4.10 ± 0.33	4.04 ± 0.29	0.256
Hemoglobin (g/dL)	11.68 ± 2.58	11.71 ± 2.03	11.64 ± 3.17	0.870
CRP (mg/dL)	0.13 (0.05–1.20)	0.06 (0.05–1.12)	0.34 (0.08–163)	0.012 *
ARB use, *n* (%)	88 (56.1)	46 (51.7)	42 (61.8)	0.207
β–blocker use, *n* (%)	50 (31.8)	25 (28.1)	25 (36.8)	0.248
CCB use, *n* (%)	60 (38.2)	31 (34.8)	29 (42.6)	0.318
α–adrenergic blocker use, *n* (%)	21 (13.4)	10 (11.2)	11 (16.2)	0.368
Statin use, *n* (%)	73 (46.5)	41 (46.1)	32 (47.1)	0.902
Fibrate use, *n* (%)	25 (15.9)	15 (16.9)	10 (14.7)	0.715

Values for continuous variables are given as mean ± standard deviation or median and interquartile range and tested by Student’s *t*-test or Mann–Whitney U test according to normal distribution; values are presented as number (%) and analysis was done using the chi-square test. PAS, peripheral arterial stiffness; baPWV, brachial-ankle pulse wave velocity; SBP, systolic blood pressure; DBP, diastolic blood pressure; LDL-C, low-density lipoprotein cholesterol; eGFR, estimated glomerular filtration rate; TMAO, Trimethylamine N-oxide; CRP, C-reactive protein; ARB, angiotensin-receptor blocker; CCB, calcium-channel blocker; CKD, chronic kidney disease. * *p* < 0.05 was considered statistically significant.

**Table 2:** Factors correlated with peripheral arterial stiffness of chronic kidney disease patients.

Variables	Odds Ratio	95% CI	*p* Value
Trimethylamine N-oxide, 1 μg/L	1.016	1.002–1.029	0.021 *
Age, 1 year	1.081	1.043–1.121	<0.001 *
systolic blood pressure, 1 mmHg	1.042	1.004–1.081	0.031 *
Hypertension, present	0.596	0.171–2.072	0.415
Diabetes mellitus, present	3.062	0.970–9.660	0.056
Fasting glucose, 1 mg/dL	0.998	0.987–1.009	0.710
Estimated glomerular filtration rate, 1 mL/min	0.991	0.962–1.020	0.538
C-reactive protein, 1 mg/dL	1.085	0.889–1.325	0.422

Data analysis was performed using multivariate logistic regression analysis (adopted factors: diabetes mellitus, hypertension, age, systolic blood pressure, fasting glucose, estimated glomerular filtration rate, C-reactive protein and trimethylamine N-oxide). CI, confidence interval. * *p* < 0.05 was considered statistically significant.

**Table 3:** Correlation between TMAO, left baPWV, and right baPWV, with clinical variables of chronic kidney disease stage 3–5 patients.

Variables		TMAO	Left PWV	Right PWV	Age	BMI	eGFR	CRP	Glucose	LDL–C	TG	SBP	DBP
TMAO	*r* *p*		0.256 0.001 *	0.267 0.001 *	0.219 0.006 *	−0.098 0.222	−0.442 <0.001 *	0.261 0.001 *	−0.015 0.856	−0.082 0.305	−0.079 0.325	0.029 0.716	−0.049 0.546
Left PWV	*r* *p*	0.256 0.001 *		0.913 <0.001 *	0.453 <0.001 *	−0.013 0.870	−0.147 0.066	0.182 0.022 *	0.132 0.100	0.038 0.637	0.035 0.666	0.328 <0.001 *	0.103 0.200
Right PWV	*r* *p*	0.267 0.001 *	0.913 <0.001 *		0.411 <0.001 *	−0.036 0.650	−0.151 0.060	0.157 0.050	0.126 0.117	0.039 0.629	0.059 0.459	0.291 < 0.001 *	0.084 0.294
Age	*r* *p*	0.219 0.006 *	0.453 <0.001 *	0.411 <0.001 *		−0.006 0.941	−0.110 0.171	0.147 0.066	0.013 0.867	−0.024 0.767	−0.129 0.109	0.041 0.611	−0.346 <0.001 *
BMI	*r* *p*	−0.098 0.222	−0.013 0.870	−0.036 0.650	−0.006 0.941		0.166 0.038	−0.072 0.373	0.125 0.118	0.028 0.724	0.128 0.109	0.221 0.006 *	0.127 0.113
eGFR	*r* *p*	−0.442 <0.001 *	−0.147 0.066	−0.151 0.060	−0.110 0.171	0.166 0.038 *		−0.185 0.021 *	0.126 0.116	0.088 0.270	−0.033 0.680	−0.184 0.021 *	−0.058 0.472
CRP	*r* *p*	0.261 0.001 *	0.182 0.022 *	0.157 0.050	0.147 0.066	−0.072 0.373	−0.185 0.021 *		−0.034 0.673	−0.088 0.272	−0.006 0.937	0.050 0.537	−0.072 0.370
Glucose	*r* *p*	−0.015 0.856	0.132 0.100	0.126 0.117	0.013 0.867	0.125 0.118	0.126 0.116	−0.034 0.673		−0.106 0.185	0.167 0.036 *	0.028 0.729	0.031 0.697
LDL–C	*r* *p*	−0.082 0.305	0.038 0.637	0.039 0.629	−0.024 0.767	0.028 0.724	0.088 0.270	−0.088 0.272	−0.106 0.185		0.196 0.014 *	−0.028 0.730	0.120 0.133
TG	*r* *p*	−0.079 0.325	0.035 0.666	0.059 0.459	−0.129 0.109	0.128 0.109	−0.033 0.680	−0.006 0.937	0.167 0.036 *	0.196 0.014 *		−0.075 0.352	0.043 0.592
SBP	*r* *p*	0.029 0.716	0.328 <0.001 *	0.291 <0.001 *	0.041 0.611	0.221 0.006 *	−0.184 0.021 *	0.050 0.537	0.028 0.729	−0.028 0.730	−0.075 0.352		0.566 <0.001 *
DBP	*r* *p*	−0.049 0.546	0.103 0.200	0.084 0.294	−0.346 <0.001 *	0.127 0.113	−0.058 0.472	−0.072 0.370	0.031 0.697	0.120 0.133	0.043 0.592	0.566 <0.001 *	

Analysis of data was performed using Spearman correlation analysis. BMI, body mass index; eGFR, estimated glomerular filtration rate; CRP, C-reactive protein; TG, triglyceride; DBP, diastolic blood pressure; SBP, systolic blood pressure; LDL-C, low-density lipoprotein cholesterol; PWV, pulse wave velocity; TMAO, trimethylamine N-oxide. * *p* < 0.05 was considered statistically significant.

**Table 4:** Correlation between peripheral arterial stiffness and TMAO of CKD patients by subgroup analysis.

Peripheral arterial stiffness	Odds ratio	95% CI	*p* Value
Diabetes mellitus, yes	1.007	0.995–1.020	0.249
Diabetes mellitus, no	1.035	1.016–1.054	<0.001 *
Hypertension, yes	1.016	1.004–1.028	0.011 *
Hypertension, no	1.032	1.008–1.056	0.008 *
Hyperlipidemia, yes	1.014	1.003–1.025	0.017 *
Hyperlipidemia, no	1.038	1.011–1.066	0.006 *
Chronic kidney disease			
Stage 3	1.006	0.992–1.019	0.432
Stage 4	1.025	1.004–1.050	0.046 *
Stage 5	1.036	1.006–1.068	0.021 *

Analysis data was done using the univariate logistic regression analysis. CI, confidence interval. * *p* < 0.05 was considered statistically significant.

## Data Availability

The data presented in this study are available on request from the corresponding author.
